# Socio-psychological determinants of Iranian rural households' adoption of water consumption curtailment behaviors

**DOI:** 10.1038/s41598-022-17560-x

**Published:** 2022-07-29

**Authors:** Moslem Savari, Ameneh Savari Mombeni, Hamed Izadi

**Affiliations:** Department of Agricultural Extension and Education, Agricultural Sciences and Natural Resources University of Khuzestan, Mollasani, Khuzestan Iran

**Keywords:** Ecology, Evolution, Ecology, Environmental sciences, Hydrology

## Abstract

Dealing with a growing population and a shortage of drinking water is a major challenge for politicians and planners. A key factor in ensuring a sustainable water supply is water conservation at the household level, which can increase productivity and save water resources. Therefore, promoting water consumption curtailment behavior will contribute significantly to reducing the global water crisis, especially in arid and semi-arid regions. Water consumption curtailment behaviors depend on individuals’ encouragement to choose and adopt voluntary behaviors and cannot be enforced by any political or planning power. In order to encourage water conservation those social and psychological factors should be considered that influence individuals to participate or adopt water consumption curtailment behaviors. Therefore, the study of factors influencing rural households' water consumption curtailment behaviors is of great importance. This study aimed to describe the socio-psychological factors influencing water consumption curtailment behaviors among rural households in southwestern Iran. The extended theory of planned behavior (ETPB) was used as a theoretical framework in this study along with descriptive norms (DN), moral norms (MN), habits, and justification. Data were collected using a questionnaire and analyzed using structural equation modeling (SEM). The results showed that ETPB can explain 35% and 54% of intention and water consumption curtailment behaviors among rural households in Iran. Our findings may assist policymakers in reducing domestic water consumption.

## Introduction

Water is not only a vital resource for agriculture but also important for the economic and social development of various regions^1,2^ and also is intimately connected with the preservation of human generations^3^. The World Economic Forum has identified water crises as one of the top five global threats. Water crises, referring to a significant decline in the available quality and quantity of fresh water, resulting in harmful effects on human health and/or economic activity^4–8^. The Global Water Development in 2020 reports that water demand exceeds supply^9^. Therefore, one of the most important challenges facing humanity in the twenty-first century is the reduction in the quantity and quality of water suitable for human consumption^10,11^. In the long run, water scarcity causes the level of water in nature to fall steadily below human needs, jeopardizing human and social development^12,13^. Several factors contribute to water scarcity, including population growth^13–15^, climate change^15–19^, human impacts^16,17^, and urbanization^17^.

According to World Water Organization report in 2020, four billion people suffered severe water shortages for at least one month a year^4^. It is projected that water demand will increase by 20–30% by 2050 when domestic consumption will be higher than elsewhere, as has been seen a 60% increase in domestic water demand between 1960 and 2014^20,21^. According to World Water Organization report in 2020, every day, 2.1 billion people still wake up each morning without access to clean water. This means that millions of vulnerable families around the world do not drink, cook, or bathe with clean water^4^. The Middle East and North Africa (MENA) the most water-scarce and conflict-prone region in the world is already experiencing the acuteness of this challenge^22^. The MENA region constitutes nearly 6% of the world’s population and the annual average water availability per person in the region stands at only 1200 m^3^, almost six times less than the global average of 7000 m^3^^20^. The main reason for this increase is the rising demand for water in developing countries and its use for domestic purposes^13^.

Due to increasing water consumption and the prolongation of droughts, the availability of water in the arid and semi-arid regions of Iran has also declined sharply^23–26^. Therefore, water consumption curtailment behaviors at the household level are a necessity^4^, as water conservation is one of the most urgent global problems^5^. According to Warner et al.^27^ promoting the adoption of water conservation technologies and practices is critical to conserving water resources^27^. The purpose of water conservation is to use water more efficiently and reduce water consumption^28,29^. Motivation for water consumption curtailment behaviors can be attributed to many psychological factors, (including values, beliefs, confidence, attitudes, and emotional responses)^30^, socioeconomic factors (income, policies, and pricing of water), environmental factors (seasonal changes), and demographic factors (age and household size). However, most studies on water conservation have tended to focus on economic and technical methods^31^. Nevertheless, the mechanism of implementing water conservation through economic methods has often failed due to the complexity of human behavior^32^. Thus, sustaining water-saving conservation behavior requires encouraging people to make and accept voluntary choices and cannot be enforced by political forces^33,34^.

It is possible to conserve water quickly without great expense or infrastructure investment^35^. Various environmental problems arise due to negative human activities, so changing human behavior is very important to conserve natural resources^2,36^. Psychological factors play a fundamental role in human behavior and can also contribute to conservation actions^37^. The social and psychological factors that drive individuals to adopt water-conserving behavior need to be considered to promote water conservation^38^. Although recent research has emphasized the need to identify the factors that influence water consumption curtailment behaviors^39^, less attention has been paid to psychosocial factors^14^. Psychosocial theories and models can be used to understand water-saving behavior. These models are used to understand the variables that predict behavior so that they can be recognized and modified appropriately^6,40,41^.

### Literature review

water consumption curtailment behaviors are one of the most important issues in water demand management^42^. Water consumption curtailment behaviors refers to the correct and efficient use of water resources in other words, any plan and strategy that leads to reducing water consumption^43,44^. Water consumption curtailment behaviors fall into two general categories: water-efficiency behaviors and water curtailment behaviors^42,45,46^. Water-efficiency behaviors require the purchase of water efficiency equipment to be able to save water consumption through the purchased equipment. But water curtailment behaviors include measures that reduce water consumption such as turning off the tap while brushing, shorter baths, etc.^45^. Water consumption curtailment behaviors are highly dependent on consumers' awareness and understanding of water consumption and how to use it in daily activities^46^. Therefore, understanding the decision-making process of individuals in the application of conservation behaviors is very important^47^. Studies have shown that psychological and social drivers play an important role in water demand management^9,46,48,49^. A relatively wide range of psychosocial theories have been applied to understand water consumption curtailment behavior so far, including the Technology acceptance model (TAM)^41^, The Norm activation model (NAM)^9^, the theory of planned behavior (TPB)^39^, the health belief model (HBM)^23^, and the social cognitive theory (SCT)^49,50^. TPB is one of the most widely used psychological-social theories because it encompasses the dynamic nature of human behavior^51^. It also provides a useful framework for examining the complexities of behavior^52^ and has been more successful in explaining the variance of conservation behaviors than other theories^53^. Therefore, in this study, the theory of planned behavior (TPB) was used to identify the psychosocial factors influencing water consumption curtailment behavior in rural households in Iran.

## Theoretical framework

### Theory of planned behavior (TPB)

In recent years, many approaches and behavioral models have been proposed to study the emergence of behaviors process and the factors that influence them in various fields including environmental protection behaviors^52^. As a result researchers have been seeking variables that affect behavior for decades and have identified the factors that are most likely to influence it^54^. The TPB provides greater insight into human behavior than other socioeconomic variables^51,55,56^ and is one of the most widely used frameworks for the study of individual behavior^57^. This theory has been tested in more than 4000 studies in various fields, including educational research and environmental conservation, and is one of the most widely used theories in the social and behavioral sciences^58^. It is one of the most popular social-psychological models for understanding and predicting human behavior^59,60^.

TPB is a social psychological theory stating that actual behavior is better predicted by intention or behavioral intent^61–64^. As a predictor of actual environmental behavior, intention is a very good indicator^65–67^. According to the TPB, behavioral intention is the strongest direct indicator of actual behavior^65^. According to this theory, intention refers to the motivation or plan behind an action^68^. In other words, a person's intention shows their motivation, readiness, and willingness to perform a certain behavior^69^. Three factors determine people's intentions to act: Attitude, Subjective Norm (SN), and Perceived Behavioral Control (PBC)^52^.

Attitude plays a central role in the theory of planned behavior^70^ since attitude toward a behavior indicates the context in which a person evaluates that behavior favorably or unfavorably^68,71^. A positive attitude is necessary to influence pro-environmental behavior (e.g., conservation water)^66^. Thus, changing people's attitudes towards water consumption can lead to a reduction in water consumption^72^. Subjective norm (SN) is another variable in this theory that refers to the social pressure or influence that affects individuals when making behavioral choices^68^. SN is influenced by the behavior and words of some important people in a person's life^73^. In other words, it refers to the individuals’ perception of whether others support their behavior change^74^. Thus, neighbors' approval of water-saving practices maybe even more important than behaviors that a person defines as important^75^. Third, perceived behavioral control (PBC) refers to the individual's perception of how difficult or easy a particular behavior is^53^.

### Extended theory of planned behavior (ETPB)

While TPB has been successfully used to examine the relationship between attitude structures and intentions, it is not a comprehensive model because moral norms (MN) (another influential variable) and its direct effect on behavior are not included^4^. It is morally required to perform or refrain from performing certain actions^76,77^. Many studies have shown that MN is a factor influencing behavior, and the inclusion of MN in the TPB may in some cases increase the predictive power of the model^53,75^. MN is defined as a sense of inherent moral commitment according to one's value system^78^. Indeed MNs are internalized forms of social norms that describe a desirable and acceptable way of life^79^.

In addition to MN, another determinative factor in environmentally friendly behavior, such as the economical use of water, is the descriptive norm (DN)^5,80^. "Descriptive norm" and "perceived actions of others" or "perception of other behaviors" can be considered equivalent to each other. The perceived actions of others can be considered one of the strongest predictors of behavioral intention. In other words, the normative beliefs of reference groups can influence the behavioral intention of others^5^. The concept of DN refers to a person's attitude about how much others (important people in their life) exhibit a certain behavior^81^. Consequently, people learn not only from their own experiences but also from observing the behavior and consequences of others^82^.

**Figure 1:**
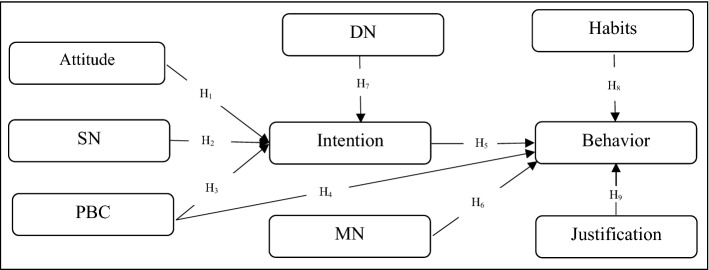
Theoretical framework.

Although the added variables can offer new insights into water demand management, their application in the field of repetitive behaviors is limited. Therefore, adding the habits variable to this theory is very important^81^. A meta-analysis by Wood and Ouellette^83^ demonstrated that regular past habits directly influence a person's future behavior independent of other variables such as attitudes, SN, intentions, and PBC^38^. In fact Ajzen^84^ suggested that habits can be integrated with other predictors of cognitive models, including attitudes, social pressure, and ease of doing an action to better predict actual behavior^81^. Human behavior that is triggered by and influenced by automated processes is called a habit^85^. Habits are typically behaviors that we perform without thinking, which are so strongly connected to the context, and do not require much mental effort so we cannot easily change^86^. If a person has a habit of conserving water, he or she is more likely to do so in the future^87^. Another variable that affects behavior is justification. Justification is primarily a rationalization of the consequences of deviant behavior (such as water waste) to protect one's blame and that of the community^88^. Despite their positive environmental knowledge and attitudes those who engage in environmentally destructive behavior justify their motivation for that behavior^88^. Justification is an internal cognitive link, which has combined functions of both preserving one's self-concept when committing acts that deviate from one's personal norm^89^, and defending oneself against possible accusations and punishments from the social environment with its formal and informal social norms^88^. Using this variable, we see that many people who waste water and do not conserve are at least partially committed to social order and conservative behavior, but what enables them to behave this way?. Justification will therefore have a negative impact on water conservation. The ETPB framework is shown in Fig. 1. Based on the discussion and arguments presented above, the following hypotheses are formulated.

- First layer: Attitude (H1), SN (H2), PBC (H3) and DN (H7) have significant effects on intentions.
- Second layer: PBC (H4), Intention (H5), MN (H6), Habits (H8) and Justification (H9) significantly affect behaviors.

## Materials and methods

### Study area

**Figure 2:**
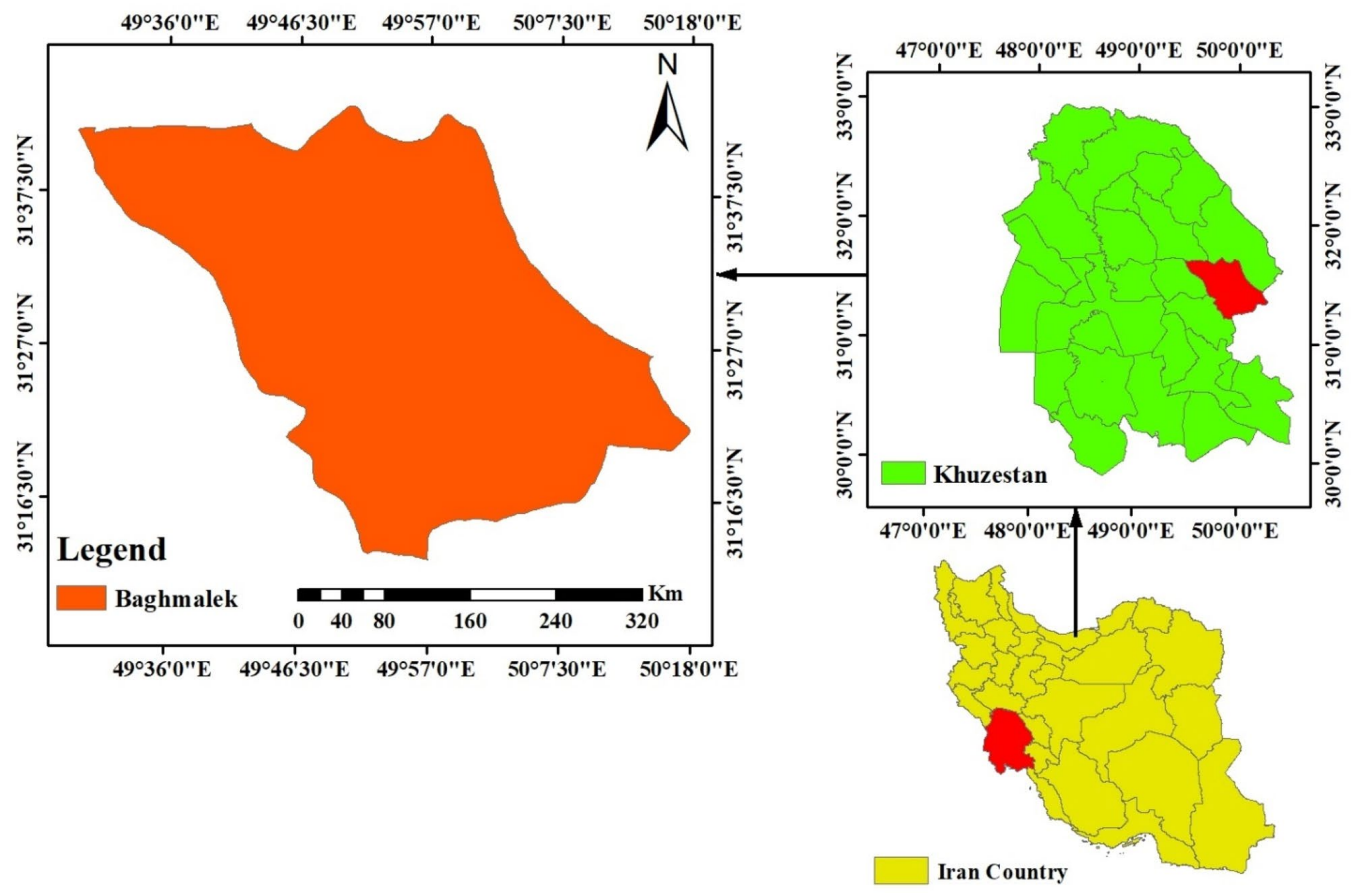
Study area.

The study was conducted in Bagh-e Malek, a city in Khuzestan province (southwest Iran) (Fig. 2). Most rural households in Khuzestan province do not practice water conservation despite being located in arid and desert areas^9^. The Bagh-e Malek County, like many other towns in Khuzestan province, is currently suffering from severe drought, and past droughts have drastically reduced rural households' access to water. Therefore studying the factors that may influence water consumption curtailment behaviors seems essential to promote a culture of water conservation and reduce water wastage in these areas.

### Study type

This practical research was conducted as a non-experimental study in terms of quantity and control degree of variables, it was a survey research regarding data collection. The statistical population of this study consisted of the rural households in Bagh-e Malek (N = 3000). Using the table of Krejcie and Morgan^90^, the sample size for the current study was 340 households using stratified random sampling method with proportional allocation. In this step, respondents were divided into two categories. The first category included those respondents who had literacy to write and read so could read the items and choose the answers based on their viewpoints, while respondents in the second category could not write and read the items of the questionnaire. Hence, the respondents in the second category were interviewed. Therefore, illiteracy did not affect the research results.

### Participants

The mean age of the respondents was 40.60 ± 11.12 S.D. years. 198 males and 142 females constituted 58.2 and 41.8% of the respondents, respectively. Regarding marital status, the results showed that 299 respondents (87.9%) were married and 41 persons (12.1%) were single. In addition, results on educational status showed that 168 (49.4%) had primary school, 54 (15.9%) had secondary school, 68 (20%) had diploma, 12 (3.5%) had postgraduate degree, 31 (1 9.9%) had bachelor's degree and 7 (2.1%) had master's degree or above. In addition, 318 households (93.5%) had their own housing, while 22 (6.5%) had rented dwellings.

### Statement

All interviewees were informed about data protection issues by the enumerators and gave their consent orally at the beginning of each interview. Informed consent was obtained from all individual participants included in the study. All materials and methods are performed in accordance with the instructions and regulations and this research has been approved by a committee at Agricultural Sciences and Natural Resources University of Khuzestan, Mollasani, Iran. All procedures performed in studies involving human participants were in accordance with the ethical standards of the institutional research committee and with the 1964 Helsinki declaration and its later amendments or comparable ethical standards.

### Measurements

Data were collected using a questionnaire that consisted of two sections. Section (i) was about the personal and professional characteristics of the respondents and section (ii) was about measuring the variables of the theoretical framework (Fig. 1) to measure Attitude (5 items), SN (5 items), PBC (6 items). DN (6 items), MN (6 items), Habits (5 items), Justification (5 items), Intention (6 items), and Behavior (7 items) were used (Table 1). A five-point Likert scale (1—very low to 5—very high) to measure the items from the household's perspective.

**Table 1:** Survey items and relevant references.

Construct	Statement	References
Attitude	I think conservation water is a good behavior	Yazdanpanah et al.^33^, Gregory and Leo^91^, Keramitsoglou and Tsagarakis^92^, Kumar Chaudhary et al.^93^
	I think it is wise to reduce wasteful consumption	
	I think it is better to use less water	
	I think using less water is beneficial	
	I think wasting water is wrong	
Subjective norm (SN)	Water conservation is often important to the people around me	Kumar Chaudhary et al.^3^, Yazdanpanah et al.^49^, Liang et al.^74^, Pradhananga et al.^94^
	People around me think i should use less water	
	Using less water will make important people happy	
	My family encourages me to save water	
	Many of the people who are important to me think water conservation is a wise decision	
Perceived behavioral control (PBC)	It is easy for me to use less water	Yazdanpanah et al.^33^, Chaudhary et al.^38^, Liang et al.^74^, Savari et al.^79^, Jorgensen et al.^86^
	I can save water	
	I can use less water	
	I know how to reduce water consumption completely	
	I am sure that it will be easy for me to use less water	
	Generally, I can save water	
Descriptive norm (DN)	I believe my family saves water	Heath and Gifford^95^, Bodimeade et al.^96^, Veisi et al.^97^
	I think most of my friends save water	
	I am sure that the people around me save water	
	I believe that most of my family thinks that wasting water is unreasonable	
	I am sure that my family thinks that wasting water is wrong	
	Wasting water is disgusting to the people around me	
Moral norm (MN)	I feel bad about wasting water	Kumar Chaudhary et al.^93^, Pradhananga et al.^94^, Botetzagias et al.^98^
	Water conservation is a matter of conscience for me	
	I feel compelled by my conscience to save water	
	I feel compelled by conscience to reduce water consumption	
	I feel uncomfortable when I observe excessive water consumption	
	I feel I have to prevent water from being wasted	
Habits	Water conservation is a habit for me	Jorgensen et al.^86^, Gregory and Leo^91^, Martínez-Espiñeira and García-Valiñas^99^
	The way my family uses water is entirely out of habit	
	I do not think about water conservation at home because I do it automatically	
	I habitually always pay attention to optimal water consumption and always do it	
	I always use water properly wherever I am because it has become a habit for me	
Justification	Due to water wastage in other areas like agriculture and factories etc., household consumption is low and saving has no meaning	Hansmann et al.^88^, Tang et al.^89^
	Many water conservation activities are small and hard to notice	
	The habit of reducing water consumption is usually forgotten when using water at home	
	My small contribution to controlling dehydration and reducing water use is not so important that I limit my work	
	Reducing water consumption is often overlooked because it has several aspects and you do not remember it while you are doing something	
Intention	I intend to reduce water consumption soon	Yazdanpanah et al.^33^, Chaudhary et al.^38^, Liang et al.^74^
	I intend to encourage others to reduce water consumption	
	I intend to reduce water consumption soon	
	I intend to model low water use soon	
	I intend to plan how to manage water consumption	
	I will try not to wastewater from now on	
Behavior	I avoid constantly opening the faucet when cleaning the garden	Chaudhary et al.^38^, Kumar Chaudhary et al.^93^, Dolnicar et al.^100^ Untaru et al.^101^
	I will not open faucet when washing dishes	
	I use the leftovers for watering house and garden plants	
	I keep the water pressure low when bathing	
	I repair broken pipes and leaks immediately	
	I turn off the water when brushing my teeth	
	I do not use tap water to wash my car	

### Validity and reliability of the research instruments

Before interviewing the rural households, a specialized team reviewed the questionnaire and modified it based on their suggestions. Besides, the index of average variance extracted (AVE) was used to evaluate the construct validity. In addition, Cronbach's alpha coefficient and composite reliability (CR) were used to evaluate reliability. If the values of AVE, CR and Cronbach's alpha are higher than 0.5, 0.6 and 0.7 respectively, the measurement model is reliable in terms of reliability and validity^102^. Reliability refers to a criterion instrument or method that produces similar results under consistent conditions. The selected items are reliable if they produce similar results under the same conditions^102^. According to Cronbach's alpha and CR values, the selected indicators were chosen accurately and accurately (Table 2). The validity indicates whether the selected measurement tool represents the feature and specification for which the instrument is designed. In other words, validity determines the extent to which the tool measures the considered attribute^102^. According to the reported amount of AVE can be said, the questionnaire's items assessed the considered features accurately (Table 2).

**Table 2:** Correlations with square roots of the AVE.

Variables	1	2	3	4	5	6	7	8	9	α	AVE	CR
Attitude	0.77^a^									0.85	0.60	0.88
SN	0.58**	0.73								0.75	0.54	0.85
PBC	0.43**	0.27**	0.74							0.89	0.56	0.88
DN	0.38**	0.28**	0.59*	0.76^a^						0.86	0.59	0.90
MN	0.45**	0.30**	0.88**	0.55**	0.73					0.84	0.53	0.87
Habits	0.43**	0.53**	0.50**	0.33**	0.51**	0.88				0.94	0.78	0.94
Justification	− 0.45**	− 0.52**	− 0.41**	− 0.29**	− 0.49**	− 0.64**	0.87			0.93	0.76	0.93
Intention	0.44**	0.20**	0.56**	0.52**	0.52**	0.34**	− 0.33**	0.86		0.95	0.75	0.95
Behavior	0.48**	0.45**	0.43**	0.28**	0.48**	0.56**	− 0.63**	0.50**	0.81	0.93	0.66	0.93

^a^The square roots of AVE estimate.

**Correlation is significant at the < 0.01 level.

### Data analysis

We used SPSS and AMOS to analyze the data in two sections of descriptive and inferential statistics. Structural equation modeling (SEM) was used to examine the factors that influence water consumption curtailment behavior and to explain the explanatory power of the research theoretical framework in predicting behavior. SEM is an important tool in the social and behavioral sciences that can be used to model theoretical concepts in terms of hidden variables and the relationship between these variables through a structural model^103^. The researchers have used this modeling tool extensively as it provides a quantitative method for testing hypotheses and unlike linear models used in traditional methods such as multiple regression estimation, it can also measure measurement error^53^. SEM consists of two levels of measurement and structural model. The Chi-square index was used to measure the fit of the model, but it is not very reliable because it depends on the sample size^102^. Therefore, RMSEA (Root Mean Square Error of Approximation), IFI (Incremental Fit Index), and CFI (Comparative Fit Index) were used in this study If RMSEA value is less than 0.08 and IFI and CFI values are more than 0.9, then the model is appropriate and the research hypotheses can be tested^46^.

### Informed consent

Informed consent was obtained from all individual participants included in the study.

## Results

### Measurement model

**Figure 3:**
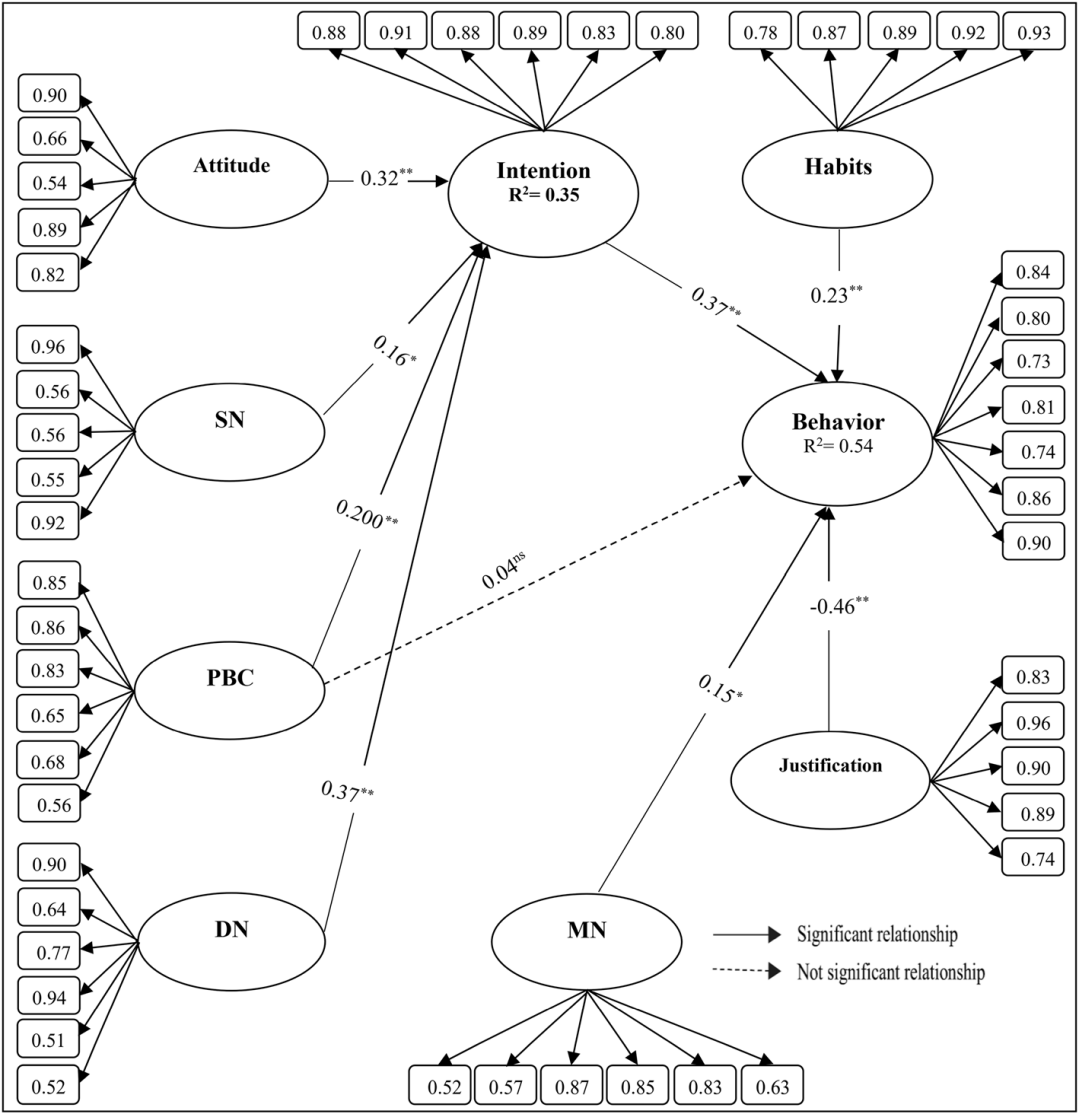
Structural path model. *Significant at the level of 0.05; **Significant at the level of 0.01; ^ns^No significant level.

Incremental indices, CFI = 0.983 and IFI = 0.982, indicated an acceptable fit of the model. Moreover, the absolute fit index RMSEA was 0.071. Since all factor loadings in Fig. 3 were higher than 0.5, the criterion of unidimensionality of the markers is confirmed^9^. Also, when examining the validity and reliability of the research instrument, the results revealed that the values of CR were higher than 0.6, Cronbach's alpha was higher than 0.7, and the index of AVE was higher than 0.5 (Table 2). Therefore it can be indicated that the measurement indicators were developed with high accuracy validity and reliability.

#### Discriminant validity

Discriminant validity: Diagnostic validity occurs when questions measuring one variable differ from questions measuring other variables. Discriminant validity is confirmed when the square roots of AVE values for all research constructs are greater than the correlation between them. In other words, the square root of the AVE value for a construct must be higher than the correlation between the construct and other constructs^104^. According to Table 2, for all constructs, the square root of the AVE value for a certain construct is greater than the correlation between that and other ones.

### Structural model

The value of χ^2^ in the structural model χ^2^(df) = 2895.289 (1089), which is statistically at the level of *P* < 0.0001. The other indices were CFI = 0.957 and IFI = 0.969 and the RMSEA value was 0.061, indicating an appropriate fit of the structural model. The results of the structural model demonstrated that it can determine 35% and 54% of water conservation intention and water consumption curtailment behaviors, respectively. Based on the results (Table 3 and Fig. 2) it can be observed that the variables justification (Beta = − 0.46, *P* < 0.001) intention (Beta = 0.37, *P* < 0.001), habits (Beta = 0.23, *P* < 0.001) and MN (Beta = 0.15, *P* < 0.001) have a direct and significant effect on the behavioral variable, with the variable justification showing the greatest effect compared to the other variables. The variables DN (Beta = 0.37, *P* < 0.001), attitude (Beta = 0.32, *P* < 0.001), PBC (Beta = 0.20, *P* < 0.001) and SN (Beta = 0.16, *P* < 0.020) have an indirect and significant effect on the behavioral variable due to intention factor. According to the obtained results, all of the predicted paths (except PBC effect) in the ETPB model were significant because the t values of all constructs were > 1.96, indicating a good fit of the ETPB model designed for fwater consumption curtailment behaviors (Table 3).

**Table 3:** The effect of forecasting variables in ETPB.

Path	Effects				
	B	β	P	t	R^2^
Attitude → intention	0.336	0.32	0.001	8.352 > 1.96	0.35
SN → intention	0.141	0.16	0.023	2.058 > 1.96	
PBC → intention	0.419	0.20	0.001	2.885 > 1.96	
DN → intention	0.322	0.37	0.001	9.684 > 1.96	
PBC → behavior	0.060	0.04	0.629	0.028 < 1.96	0.54
MN → behavior	0.216	0.15	0.042	2.114 > 1.96	
Habits → behavior	0.260	0.23	0.001	3.025 > 1.96	
Justification → behavior	0.436	0.46	0.001	12.356 > 1.96	
Intention → behavior	0.320	0.37	0.001	9.625 > 1.96	

β: Standardized factor loading; B: Non-standard factor loading, P: Significant at level (0.05 or 0.01).

## Discussion

Climate change along with population growth lead to water scarcity that is a major problem worldwide. It is therefore essential to conserve water resources and prevent future shortages^14^. Adopting water consumption curtailment behaviors leads to a considerable increases in optimal water consumption and prevents excessive water wastage. By water consumption curtailment, we can help preserve this valuable resource for future generations. In addition, the application of water consumption curtailment behavior leads to environmental protection and an increase in the quantity and quality of groundwater^46^. Hence, adopting water consumption curtailment behaviors is essential to reduce the negative impact of water scarcity in households. Understanding the factors that influence water consumption curtailment behavior can assist governments and policymakers to improve this behavior and prevent the waste of water resources. The purpose of this study was to identify the social and psychological factors that influence household water consumption curtailment behaviors. TPB was used by expanding it with additional variables like MN, DN, habitat and Justification was used in this study to investigate the factors affecting water conservation in rural households and SEM, to analyze the data and test the research hypotheses. The innovations of the current study are remarkable in two aspects that have received less attention in previous research. (i) The extended TPB model in this research was not used in any of the previous studies (four variables added simultaneously). (ii) The study area is strongly affected by water shortage and its effects, however this kind of research has not been done in this area. The results are arranged in accordance with the research hypotheses.

The results showed that attitude can effectively predict water consumption curtailment behavior through intentions. Thus, the H1 hypothesis was confirmed. Studies Warner and Diaz^4^, Kumar Chaudhary et al.^27^, Russell and Knoeri^39^, Shahangian et al.^48^, Wang et al.^52^, Lam^55^, Zhong et al.^70^, Liang et al.^74^, Maduku^81^, Clark and Finley^105^, is consistent with our findings. This result can be explained by the fact that consumers' attitude influences water consumption curtailment behavior and associated savings. This is because attitude refers to a person's evaluation of the behavior^74^. Therefore, people who have a positive attitude towards saving water are more likely to do so^65^. Although the financial benefits of saving water seem to be important to the household, the study of De Dominicis et al.^106^ Suggested that social incentives are more effective than financial incentives in adopting environmentally friendly behaviors. Consequently, changing people's attitudes through social activities and training courses in water conservation activities may be effective in this case, as long as people are not aware of the significance of the behavior and its consequences, attitudes, and beliefs will not change.

According to the H2 hypothesis, SN was effective in predicting water consumption curtailment behaviors through intentions. The results of the study Warner and Diaz^4^, Savari et al.^9^, Yazdanpanah et al.^33^, Shahangian et al.^48^, Lam^55^, Liang et al.^74^, Maduku^81^, support our finding. The results of the H2 hypothesis indicate that individuals are influenced by various people in society such as parents, spouses, local leaders, family, etc., and whether or not they perform a behavior may be affected by their influence or pressure. This means that a person's intention is determined by the desires of others^99^. Therefore, if the behavior of saving water and using water sparingly is accepted by people with high social status, it will be effective for others to implement the conservation behavior^4^.

In addition, the results showed that PBC influenced water consumption curtailment behaviors through behavioral intention (confirming Hypothesis 3). However, PBC did not directly influence water consumption curtailment behavior (which refutes hypothesis 4). The research results of Warner and Diaz^4^, Savari et al.^41^, Ajzen^55^, Liang et al.^74^, Jorgensen et al.^86^, Wang et al.^107^ support our finding. According to the PBC, motivation is influenced by the appraisal of the difficulty of a particular behavior and the success in accomplishing the task. Individuals who have greater self-confidence and perceive less difficulty in using protective measures are more likely to adopt them^41^. When a person has a strong control belief regarding the existence of factors that facilitate a behavior they will have high perceived control over a behavior. While if a person does not have strong control beliefs, they will show a low sense of control, which will prevent them from taking action^107^. Consequently, rural households need to be educated on how to conserve water and use it effectively to prevent water loss and strengthen their capabilities.

According to the H5 hypothesis, intention was effective in predicting water consumption curtailment behavior. The studies of Yazdanpanah et al.^33^, Shahangian et al.^48^, Popa et al.^61^, Tam^62^, Marcos et al.^67^, Maduku^81^, Wang et al.^107^ support our finding. Intention to conserve water refers to a person's commitment to engage in conservation activities and specifically to conserve water^61^. Intention has always been key to understanding behavior and is one of the best predictors of actual behavior^60^. Consequently households that have a strong desire to conserve and protect water are more likely to engage in protective behaviors in the future.

The results of hypothesis H6 indicated that MN was one of the effective factors for water consumption curtailment behavior in rural households in Iran. The research results of Warner and Diaz^4^, Savari et al.^9^, Aslam et al.^13^, Yazdanpanah et al.^33^, Shahangian et al.^48^, Gkargkavouzi et al.^75^, Kumar Chaudhary et al.^93^ agree with this result. MN is the moral force and imperative to perform a certain behavior^4^. Consequently, people with higher moral commitment are more likely to behave environmentally conscious and save water^9^. According to the importance of MN, more rural households should adhere to ethical principles in water management to build a foundation for environmentally friendly and protective behaviors. Similarly, Yazdanpanah et al.^33^ claim that it may be helpful to consider MN in the context of water consumption curtailment behaviors, especially in Islamic countries such as Iran, where water is considered sacred and a gift from God. Therefore, ethical norms can be an important part of water resources strategies and management in Iran, especially in rural areas.

DN was another factor that influenced water consumption curtailment behaviors in rural households. This factor affected water consumption curtailment behaviors indirectly and through behavioral intention. This result confirms hypothesis H7. The results of studies Warner^5^, Warner et al.^27^, van Valkengoed and Steg^80^, accord with this finding. DN exhibits an understanding of the prevalence of a behavior^108^. Thus DN implies that people learn not only from their own experiences but also from the results and experiences of others and that they attach importance to the actions and behaviors of others^80^. This could be explained by the fact that when people observe many of their relatives, friends and acquaintances conserving water consumption, their motivation increases to do the same. Therefore, successful examples of water use reduction in rural communities should be identified, promoted, and supported to encourage other individuals and rural households to implement similar practices.

Another factor influencing water consumption curtailment behavior in rural households is a habit (confirming hypothesis 8). Other researchers^39,81,86,87,91,99^, have reported similar findings. A person's behavior can sometimes be spontaneous, out of habit, and without conscious thought and consideration^81^. According to psychological research, people's behavior depends heavily on their habits, and habits play a crucial role in the adoption of protective behaviors^109^. In this way, individuals often consider their misbehavior as a long-term habit that they do not want to change. Indeed, water waste is a consequence of persistent water consumption habits^99^. Developing water-saving habits can influence individuals' attitudes. ​

According to the H9 hypothesis, justification was negative effective in predicting water consumption curtailment behaviors. The results of the study Hansmann et al.^88^, Tang et al.^89^ support our finding. According to this hypothesis, the more a water-wasting behavior is justified, the less protective it is, as justifications help to explain deviant behavior (such as excessive water consumption)^88^. Among human daily activities, there are behaviors that can be changed to prevent water wastage on a daily basis. While people consider different reasons and justifications to convince themselves that this change in behavior and the way they or even their family use water cannot have a significant effect on reducing water consumption and improving dehydration. Despite this mindset, the reality is quite different and they are probably unaware of how important their behavior and that of those around them is in reducing consumption.

## Policy implication

In general, based on the research results, the following three policies are proposed for the use of water consumption curtailment behaviors among rural household. Adopting these policies can affect the use of water consumption curtailment behaviors among rural household.

(i) Development of incentive-motivational activities: In order to apply water consumption behaviors, households with lower water consumption need to be identified and supported by the government (for example, purchase of equipment to reduce water consumption) so that other rural households have the incentive to reduce water consumption.
(ii) Development of water protection norms: The findings of this study showed that subject and moral norms have a significant effect on water consumption curtailment. Therefore to increase the likelihood that water consumption curtailment behaviors will be adopted it is suggested that information and education programs on optimal water use be implemented by people with high social influence.
(iii) Increasing awareness: Many rural households are not fully aware of the consequences of their behavior, and according to daily habits, part of the water is wasted every day. It is suggested to make society aware of the global drought by highlighting the dangers of dehydration and drought and involving more knowledgeable people and professionals through local radio and television. If we take these measures, we can see all sections of society conserve water without irrational justifications and excuse.

## Conclusions

In this study, TPB was used by expanding it with additional variables like habit, MN, DN, habitat and Justification was used to investigate the factors affecting water consumption curtailment behaviors that was relatively successful as it explained 54% of water consumption curtailment behaviors. Moreover, among the variables studied, the factors of justification, DN, and habits exhibited the greatest influence on water consumption curtailment behaviors. In addition, the results showed that the developed theory of TPB was able to explain 35% of the variance of intention and the most important influential variables were DN and attitude. Moreover, despite its important results, two critical limitations should be noted: first, the variance has not yet been partially explained. Therefore, it is necessary to further improve the explanatory power of the model by reviewing the research literature in more detail and identifying important variables. Second, the ETPB model was used in this research. Therefore, it is better to determine its explanatory power concerning water consumption curtailment behaviors by testing other psychological models to contribute to the literature in this area.

## Data Availability

The datasets generated during and/or analyzed during the current study are available from the corresponding author on reason able request.
